# Graph-Based Pan-Genome Reveals the Pattern of Deleterious Mutations during the Domestication of *Saccharomyces cerevisiae*

**DOI:** 10.3390/jof10080575

**Published:** 2024-08-14

**Authors:** Guotao Chen, Guohui Shi, Yi Dai, Ruilin Zhao, Qi Wu

**Affiliations:** 1 State Key Laboratory of Mycology, Institute of Microbiology, Chinese Academy of Sciences, Beijing 100101, China; chengt@im.ac.cn (G.C.); shigh@im.ac.cn (G.S.); daiyi1993@hotmail.com (Y.D.); 2 University of the Chinese Academy of Sciences, Beijing 100049, China

**Keywords:** deleterious mutations, *Saccharomyces cerevisiae*, domestication, heterozygosity, graph-based pan-genome

## Abstract

The “cost of domestication” hypothesis suggests that the domestication of wild species increases the number, frequency, and/or proportion of deleterious genetic variants, potentially reducing their fitness in the wild. While extensively studied in domesticated species, this phenomenon remains understudied in fungi. Here, we used *Saccharomyces cerevisiae*, the world’s oldest domesticated fungus, as a model to investigate the genomic characteristics of deleterious variants arising from fungal domestication. Employing a graph-based pan-genome approach, we identified 1,297,761 single nucleotide polymorphisms (SNPs), 278,147 insertion/deletion events (indels; <30 bp), and 19,967 non-redundant structural variants (SVs; ≥30 bp) across 687 S. cerevisiae isolates. Comparing these variants with synonymous SNPs (sSNPs) as neutral controls, we found that the majority of the derived nonsynonymous SNPs (nSNPs), indels, and SVs were deleterious. Heterozygosity was positively correlated with the impact of deleterious SNPs, suggesting a role of genetic diversity in mitigating their effects. The domesticated isolates exhibited a higher additive burden of deleterious SNPs (dSNPs) than the wild isolates, but a lower burden of indels and SVs. Moreover, the domesticated *S. cerevisiae* showed reduced rates of adaptive evolution relative to the wild *S. cerevisiae*. In summary, deleterious variants tend to be heterozygous, which may mitigate their harmful effects, but they also constrain breeding potential. Addressing deleterious alleles and minimizing the genetic load are crucial considerations for future *S. cerevisiae* breeding efforts.

## 1. Introduction

All organisms carry a certain number of deleterious mutations in their genomes. Deleterious mutations cause loss or alteration of normal gene function, leading to reductions in organismal fitness [1]. The majority of these deleterious mutations are detrimental and recessive, whereas only a few are dominant or recessive lethal [2]. Although most of these have a negative effect under natural conditions, some may generate a desirable phenotype under domestication and can be retained by artificial selection [3]. Extensive studies have reported that domesticated species, such as horses [4], dogs [5], rice [6], sheep [7], tomatoes [8], and yeast [9,10], are burdened by many more deleterious mutations than their wild relatives, which is known as the “cost of domestication” hypothesis [6,11]. To reduce genetic loads, a key intriguing pattern emerged showing that the deleterious variants commonly exhibit higher heterozygosity when compared to variants having other genomic impacts [12,13]. A plausible explanation could be that some deleterious variants are recessive and can be masked in a heterozygous state to alleviate their harmful effects [13]. The dominance hypothesis suggests that when individuals carry slightly deleterious recessive alleles, they can complement each other in heterozygous combinations. This complementation tends to contribute more to genetic variation within populations rather than causing significant genetic differences between populations [14]. As a result, heterozygous individuals may exhibit increased vigor compared to homozygous individuals for these alleles [9]. However, high levels of heterozygous deleterious variants could considerably constrain their breeding potential. When seeds are selfed or crossed, a large proportion of deleterious variants are homozygous and cause severe inbreeding depression [15,16]. Thus, understanding the allele state of deleterious variants and reducing the genetic load in the genomes accumulated during domestication is important for breeding.

Baker’s yeast, *Saccharomyces cerevisiae,* is considered to be one of the earliest domesticated species. Evidence of fermented wine-like beverage production dates back to the Neolithic period, approximately 9000 years ago, in China [17]. The budding yeast *S. cerevisiae* has been used worldwide for baking, brewing, distilling, winemaking, and bioethanol production [18,19]. Originating from ancestral interspecies hybridization followed by whole genome duplication and reduction, domesticated *S. cerevisiae* exhibits distinctive patterns of demographic history and genome evolution characterized by typical domestication signatures, such as historical bottlenecks, increased rates of unbalanced rearrangements, polyploidies, and aneuploidies [20,21,22,23]. These led to extensive genomic changes associated with the ecological adaptation of domesticated *S. cerevisiae*, including heterozygosity, gene contraction or expansion, SNP accumulation, and acquisition of foreign genes through horizontal gene transfer (HGT) or introgression [24,25,26,27,28]. However, few studies have focused on the “cost of domestication” of *S. cerevisiae*. Only two studies have identified the accumulation of deleterious SNPs (dSNPs) in beer strains and the West African population [25,29]. Furthermore, certain adaptations found in wild *S. cerevisiae* were reduced in the domesticated strains. For example, Duan et al. [24] showed that the sporulation rate of wild populations was significantly higher than that of domesticated populations. The majority of the wild strains sporulate well, whereas most domesticated strains fail to sporulate. Furthermore, more than 95% of the ascospores formed by the wild strains were viable, but less than 20% of the ascospores formed by the domesticated strains were viable [30]. Nevertheless, comprehensive studies on the genome-wide patterns of deleterious mutations in wild-to-domesticated *S. cerevisiae* are needed. 

This study aimed to investigate the genomic patterns of deleterious mutations during the domestication of *S. cerevisiae*. To obtain comprehensive variation information, we constructed a pan-genome graph using 41 diverse accessions representing major subpopulations of *S. cerevisiae* worldwide. We studied single nucleotide polymorphisms (SNPs), insertion/deletion mutations (indels), and structural variations (SVs) from a set of 231 wild isolates, 456 domesticated isolates (180 isolates from solid-state fermentation (SSF), and 276 isolates from liquid-state fermentation (LSF)). We then inferred the strength of selection against different types of variants, identified assumed deleterious mutations, and investigated the patterns of deleterious mutations during *S. cerevisiae* domestication. In addition, we examined the distribution of deleterious variants relative to genome-wide recombination rates and the locations of the putative selective sweeps.

## 2. Materials and Methods

### 2.1. Source of S. cerevisiae Genome Data

We first downloaded high-quality genomes of 41 isolates of *S. cerevisiae* from the National Center for Biotechnology Information (NCBI) and assembled them based on third-generation sequencing to construct a pan-genome (Table S1). Among these 41 isolates, 17 isolates were sourced from wild isolates and 24 isolates were isolated from ancient fermentation processes. For further population genetic analyses, paired-end reads of 687 *S. cerevisiae* isolates were downloaded from NCBI (Table S2). These isolates represented different lineages of *S. cerevisiae* with different ecological and geographic origins [24,25,27,31,32]. Based on the source, these isolates were divided into 3 groups, including 231 wild isolates derived from primary forest, secondary forest, and fruit, 180 isolates associated with the solid-state fermentation (SSF) process, including Mantou, Baijiu, Huangjiu, and Qingkejiu, and 276 isolates associated with the liquid-state fermentation (LSF) process, including beer and wine. The ploidies are also provided in Table S2, and the majority of the isolates have a diploid genome (Table S2). Detailed information for each isolate can be found in Table S2.

### 2.2. Graph-Based Pan-Genome Construction and Population-Scale Genotyping

To obtain comprehensive variation information, particularly the structural variant (SV) information, of *S. cerevisiae*, we used the minigraph-cactus pan-genome pipeline [33] to construct a pan-genome graph of 41 TGS-based (third-generation sequencing-based) *S. cerevisiae* genomes (Table S1) and reference genome S288C. Briefly, we first used Minigraph v0.19 [34] to construct a rough graph with only SVs larger than 50 bp across the 42 genomes. We then used the minigraph-cactus pipeline to add the SNP-level variants to the graph. The repeating regions in the graph were identified and soft-masked using RepeatMasker to exclude the influence of highly repetitive sequences on the subsequent analysis. The assemblies were remapped to a minigraph to produce exact alignments between the contigs of the input assembly and the minigraph node sequences. Subsequently, we split the graph assigned to the contigs into different chromosomes, and applied Cactus v2.1.1 base alignment separately; the output HAL files were converted to the vg format with hal2vg v2.1 (https://github.com/ComparativeGenomicsToolkit/hal2vg, accessed on 8 November 2022.). Paths larger than 10 kb that did not align with the underlying graph were removed, and GFAffix (https://github.com/marschall-lab/GFAffix, accessed on 26 September 2022) was used to normalize the graph. Finally, the graphs of the chromosomes were combined into a whole-genome graph, indexed, and exported to VCF using the vg toolkit for vg toolkit v1.40.0 [35]. 

Illumina paired-end reads of the 687 *S. cerevisiae* isolates (Table S2) were subsequently mapped against the graph genome, alignments in the GAM format were generated, and an augmented GAM reads file was produced using ‘vg augment’. Alignments with mapping quality <5 and breakpoints with coverage < 10% were excluded. A compressed coverage index was calculated using ‘vg pack’ and snarls were generated using ‘vg snarls’. The variants genotyped for each isolate were produced using ‘vg call’ with the ‘-v’ parameter. These variants included SNP, short indels (<30 bp), and SVs (≥30 bp). The SVs included insertions (INSs) and deletions (DELs). Based on the S288C genome, SnpEff v4.3 was used to annotate the identified variants [36] and the nonsynonymous and synonymous SNP sites (nSNPs and sSNPs).

### 2.3. Phylogenetic Reconstruction 

To determine the relationships among the 687 *S. cerevisiae* isolates, we utilized a comprehensive VCF file containing all isolates and retained all the variants. The pairwise distances between each pair of isolates were computed using VCF2Dis v.1.42 (https://github.com/BGI-shenzhen/VCF2Dis, accessed on 25 July 2022). Subsequently, a phylogenetic tree in the Newick format was inferred from these p-distance matrices using FastME ver. 2.0 [37]. The resulting tree was visualized using iTOL, which provided a clear depiction of evolutionary relationships within the *S. cerevisiae* isolates.

### 2.4. Genetic Diversity Analyses

Genetic diversity (*π*) in a 50 kb slide window was calculated using VCFtools. The top 1% of the regions with high and low *π* values were consistent with the hypervariable and conservative regions.

### 2.5. Distribution of Fitness Effects of Different Mutations

We applied the newly developed maximum likelihood framework polydfe v2 [38] to infer the distribution of fitness effects (DFE) of both the deleterious and beneficial mutations based on polymorphism data and the proportion of adaptive variation (*α*). In these analyses, we used information from the sSNPs as the neutral reference, and the unfolded site frequency spectrum (SFS) of the sSNPs, nSNPs, indels, DELs, and INSs for the three groups (wild, SSF, and LSF) was computed using the lineage of the root of the phylogenetic tree (CHN-IX/TW-1) as the outgroup. To decrease the parameters to be estimated and visualized, we projected the SFS of the 3 groups to a sample size of 15 using easySFS (https://github.com/isaacovercast/easySFS, accessed on 27 March 2023). The standard deviation was estimated by analyzing 20 bootstrap replicates of the SFS. 

### 2.6. Mutational Load Estimation

To predict the functional effects of variants, we used Sorting Intolerant from Tolerant 4G (SIFT_4G) [39] to annotate the SNP dataset. To create an *S. cerevisiae* database, uniref90 (https://www.uniprot.org/, download date: 1 March 2022) was used as a reference protein set. The annotation of S288C was downloaded from the NCBI database. The yeast SIFT_4G database was constructed using the SIFT4G_Create_Genomic_DB implemented in SIFT 4G. The SIFT scores ranged from 0 to 1, and any nonsynonymous position with a SIFT score <0.05 was considered putatively deleterious. To reduce the effects of reference bias, predictions of deleterious variants were inferred using ancestral (rather than reference) variants. For indels and SVs, the number of derived alleles was calculated for each variant type. The mutational load for each variation type was calculated using an additive (2 × homozygous variants + number of heterozygous variants) [6,40].

### 2.7. Recombination

To estimate the recombination rate along the *S. cerevisiae* genome, we calculated the population-scaled recombination rate for each 50 kb non-overlapping sliding window across the three *S. cerevisiae* groups. We phased each chromosome according to the unlinked SNPs of 687 *S. cerevisiae* isolates using beagle [41]. Then, we used FastEPRR v2 [42] to estimate the recombination rate (Rho) per 50 kb windows by combining all species. FastEPRR is a widely used R package for rapid and accurate estimation of population recombination rates from DNA polymorphisms. To investigate the relationship between deleterious mutations and the recombination rate, we calculated the number of deleterious mutations at each 50 kb non-overlapping sliding window. 

### 2.8. Detection of Signatures of Selection Using F_ST_

The population fixation index (*F*_ST_) was calculated using VCFtools (--fst-window-size 10,000 --fst-window-step 1000) [43], with the sliding window size set to 10 kb and the window step set to 1 kb. A strong selection signal within the selected region between the groups was obtained and visualized by drawing scatter plots of the origin. The top 1% of the genome-wide *F*_ST_ values were considered potential selected scan regions (SS).

### 2.9. Functional Enrichment Analyses

To gain deeper insights into the outlier genes identified within the hypervariable, conservative, and SS regions. We used ANNOVAR [44] for gene annotation and Metascape v1.0 [45] for functional enrichment analyses. 

### 2.10. Statistical Analysis

Standard statistical analyses were performed in the R project (v3.3.1) [46], and all the statistical significance tests included two-sided Student’s *t*-tests for paired analyses.

## 3. Results

### 3.1. Graph-Based Pan-Genome Construction Using 41 S. cerevisiae Genomes

**Figure 1:**
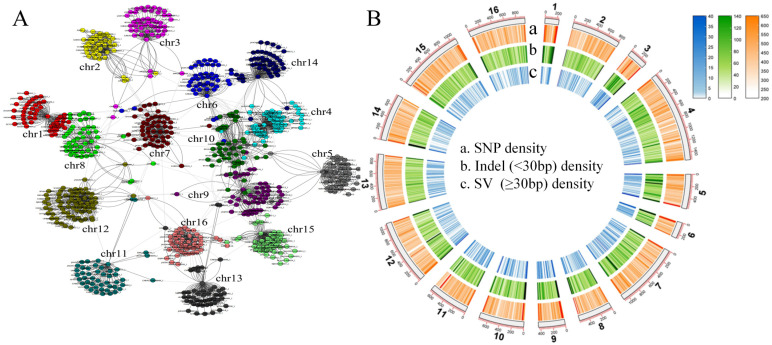
Pan-genomic landscape based on 41 third-generation genomes and the reference genome, S288C. (**A**) Network diagram of inter-chromosomal recombination relationships in 41 *S. cerevisiae* strains. (**B**) Pan-genome variation landscapes.

**Figure 2:**
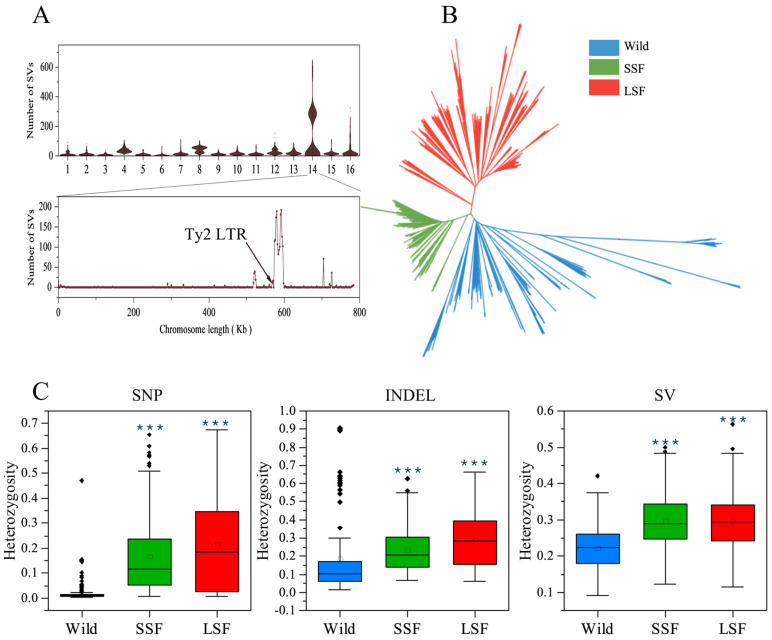
Genomic landscape of *S. cerevisiae* variation. (**A**) Distribution of structural variations across chromosomes in each strain. (**B**) NJ tree of 687 isolates based on the presence or absence of all the variants. (**C**) Ratio of heterozygous variations in the wild, SSF, and LSF groups. The middle bars represent the median, while the bottom and top of each box represent the 25th and 75th percentiles, respectively, and the whiskers extend to 1.5 times the interquartile range. Dots are outliers. *** *p* = 0.001 (*t*-test).

To capture the complete genetic diversity within a species and reduce the bias in genetic analysis inherent in using a single reference genome (S288C), we collected another 41 long-read sequencing haploid assembled genomes of *S. cerevisiae* (11.6–12.8 Mb; Table S1) to construct a pan-genome graph. The determined *S. cerevisiae* pan-genome size was 30.0 Mb, which was more than twice the size of the reference genome, S288C (12.4 Mb). Further analysis revealed many inter-chromosomal rearrangements and fusions across the 41 *S. cerevisiae* genomes (Figure 1B). Using the S288C genome as the reference skeleton, the pan-genome contained 578,129 SNPs, 130,298 small indels, and 12,977 SVs. The density of SVs and short indels at the end of the chromosome was higher than that in other regions (Figure 2B).

### 3.2. Population-Wide Variant Analyses in 687 S. cerevisiae Strains

We then mapped short-read sequencing data for 687 *S. cerevisiae* isolates collected from various environments and countries to a pan-genome and obtained 1,297,761 high-quality SNPs, 278,147 indels, and 19,967 non-redundant SVs (INSs: 7898 and DELs: 9367). Most of these variants were present at very low frequencies, with 96% having a minor allele frequency (MAF) < 0.05. Interestingly, the number of SVs in the wild, LSF, and SSF groups divided into two clusters (Figure S1). Further analyses revealed that this phenomenon was due to the presence or absence of variants of the Ty2 LTR on chromosome 14, which resulted in a difference in the number of SVs (Figure 2A). To eliminate this interference, 6038 SVs in this region were excluded from subsequent analyses. Subsequently, we used the present/absent variants to construct a phylogenetic tree. In the phylogenetic tree, the wild and the domesticated populations were clearly separated. The domesticated strains were clustered into two major groups associated mainly with liquid- and solid-state fermentation, respectively (Figure 2B). 

Genetic diversity in a 50 kb slide window was calculated using the present/absent variants in the LSF and SSF groups (Figure S3A,B). After annotation, we found that in the domesticated strains, the genes related to sugar metabolism showed higher mutation rates, such as fructose transmembrane transport (GO:0015755), maltose metabolic process (GO:0000023), fructose metabolic process (GO:0006000), and sucrose catabolic process (GO:0005987), and transport pathways, such as transmembrane transport (GO:0055085), siderophore transport (GO:0015891), inorganic anion transmembrane transport (GO:0098661), amino acid transmembrane transport (GO:0003333), and siderophore transport (GO:0015891) (Figure S3C,D). Conversely, in the conservative regions, the enriched pathways were mainly related to cellular processes such as retrotransposition (GO:0032197), translational elongation (GO:0006414), and positive regulation of ATP-dependent activity (GO:0032781) (Figure S3E,F). 

The SNP sites in the wild isolates were almost homozygous (Figure 2C), with an average ratio of heterozygous sites of 0.016 among all the variants, which was lower than that in the SSF (0.17) and LSF (0.21) groups (Table S2). In contrast to the SNPs, the ratios of the heterozygous indels and SVs were obviously increased in the wild isolates, and the average ratio of heterozygous sites among the total variants in each isolate increased to 0.18 and 0.22, respectively (Table S2). In particular, the heterozygous indels in the isolates from the Fushan Botanical Garden (Taiwan, Yilan, Yuanshan) accounted for about 0.8 of the total identified indels, and the ratio of heterozygous SVs in some wild isolates from secondary forests, orchards, fruit, and oak also reached 0.8 (Table S2). The mean heterozygous indels and SVs were 0.24 and 0.30 in the SSF group, and 0.30 and 0.30 in the LSF group, respectively, which were higher than that in the wild isolates (*p* < 0.05) (Figure 2C).

### 3.3. An Integrative Comparison among Wild, SSF, and LSF Isolates

**Figure 3:**
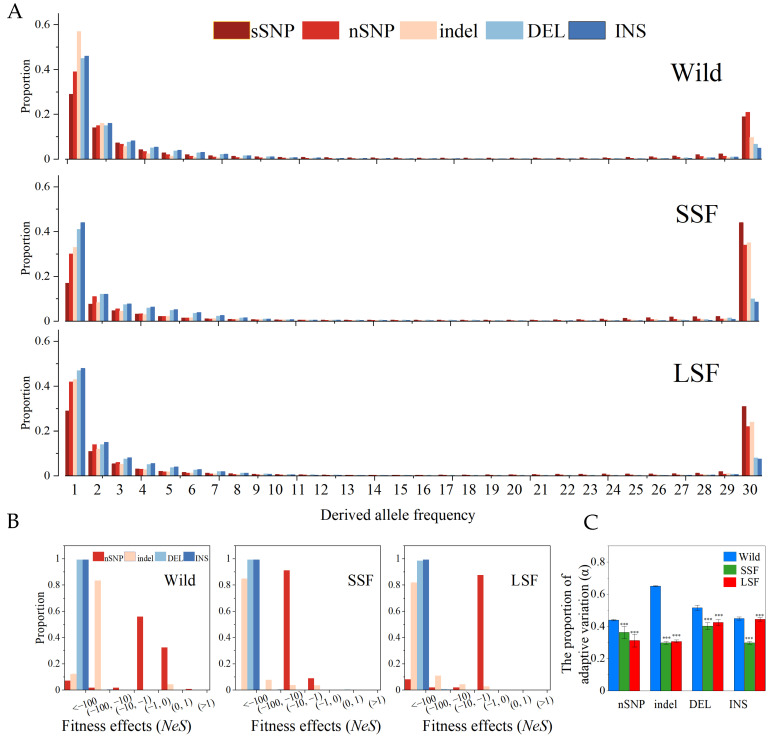
SFS spectra, fitness distribution, and proportion of adaptive variation (α) in the wild, SSF, and LSF groups, respectively. (**A**) Shows the unfolded SFS spectra of nSNPs, indels, and SVs (including INS/DEL) in wild, SSF strains, and LSF strains. (**B**) Displays the inferred fitness effects distribution (*Nes*) of nSNPs, indels, and SVs in wild, SSF strains, and LSF strains. (**C**) Proportion of adaptive variation (α) in the wild, SSF, and LSF strains. Error bars represent mean ± 95% CI. Dots are outliers. *** *p* = 0.001 (*t*-test).

We also calculated the unfolded site frequency spectra (SFS) of the three groups (Figure 3A). Each SFS included two SV types (INS and DEL) along with sSNPs, nSNPs, and indels. The proportion of fixed variants in the domesticated group was higher than that in the wild group (Figure 3A), indicating that domesticated *S. cerevisiae* might have undergone a severe domestication bottleneck, which can dramatically alter population frequencies. In all three groups, the proportion of fixed SVs was lower than that of the fixed sSNPs, nSNPs, and short indels. The SFS of all the groups differed significantly from that of the sSNPs in both taxa (*p* < 0.05, Kolmogorov–Smirnov, Bonferroni corrected). Assuming that the sSNPs provided a reasonable “neutral” control, the leftward shift in the SFS suggested that the SVs were predominantly deleterious or had higher mutation rates than the SNPs, such that many new events did not possess the opportunity to increase in frequency.

To quantify the strength of selection for the different types of mutations, we estimated the distribution of fitness effects from the population frequency data, using the sSNPs as a neutral control. In the three groups, the results confirmed that the nSNPs, indels, and SVs underwent strong purifying selection, and the fitness of each variation was <1 (Figure 3B). In particular, the fitness of nearly all the SVs was less than −100 in the three groups. Compared with the wild group, the nSNPs and indels had lower fitness effects in the domesticated groups than in the wild groups (Figure 3B). These inferences were consistent with the estimation of the proportion of adaptive variation (*α*). The proportion of adaptive mutations in the domesticated group was lower than that in the wild group (Figure 3C).

### 3.4. Genetic Burden of Mutations

**Figure 4:**
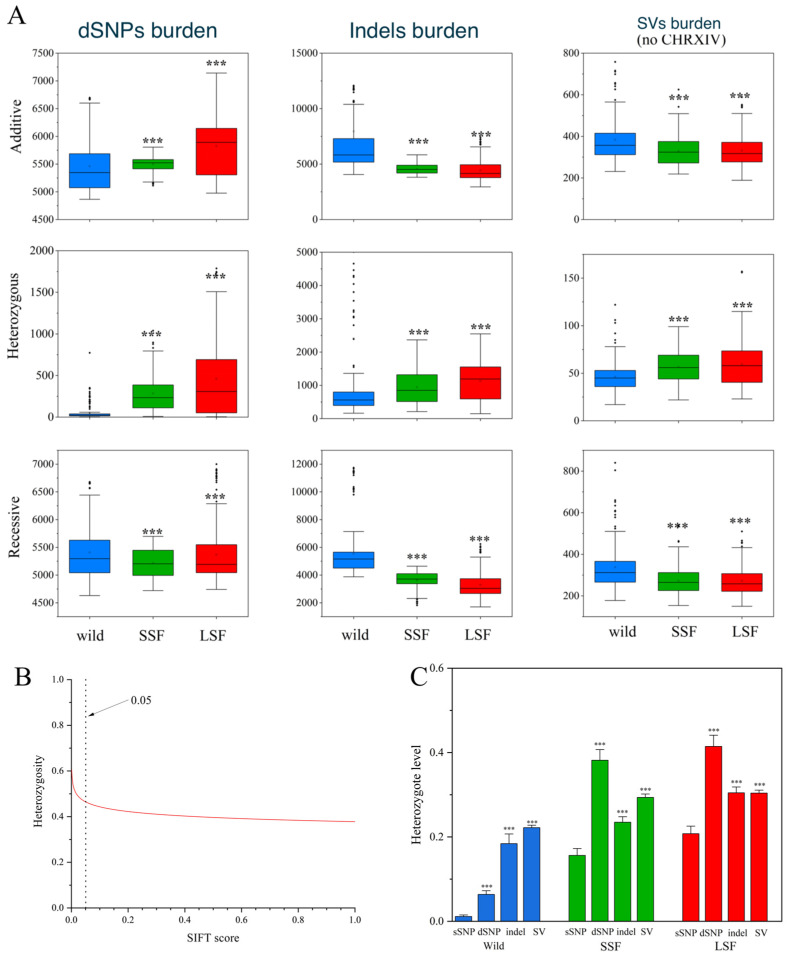
Comparison of genetic loads in *S. cerevisiae*. (**A**) Comparison of additive, heterozygous, and recessive (the number of homozygous SVs per *S. cerevisiae*) loads in wild-type, SSF, and LSF isolates. The middle bars represent the median, while the bottom and top of each box represent the 25th and 75th percentiles, respectively, and the whiskers extend to 1.5 times the interquartile range. Dots are outliers. (**B**) Correlation between levels of genomic heterozygosity and its harmfulness (estimated by SIFT v2.1). A smaller SIFT score indicates that the mutation is more likely to be deleterious. (**C**) Heterozygosity levels for different types of genic variants in the wild, LSF, and SSF groups. Error bars represent mean ± SD. *** *p* = 0.001 (*t*-test).

Here, we used SIFT scores to quantify the deleterious alleles in three groups of *S. cerevisiae* and found 95,820 deleterious SNPs (dSNPs). Based on the fitness effects, all variants other than SNPs were considered deleterious. The additive burden of dSNPs in the domesticated groups was significantly higher than that in the wild group (Figure 4A). For example, the additive burden in the LSF group increased by 9% compared to that in the wild group. However, in contrast to the dSNPs, the additive burden of variants other than SNPs decreased in both domesticated groups compared to that in the wild group. The media additive burden for the indels decreased by 22% and 29%, while the additive burden for the SVs decreased by 9% and 11% for the SSF and LSF groups, respectively (Figure 4A). Interestingly, the heterozygous burdens of the dSNPs, indels, and SVs in both domesticated groups were higher than the corresponding heterozygous burdens in the wild groups (Figure 4A). Examining the correlation relationship between each SNP heterozygosity and its SIFT score, we noticed that the levels of dSNPs heterozygosity were negatively correlated with the SIFT score (Figure 4B), suggesting that the heterozygosity level increased with the harmfulness of the dSNPs. The heterozygosity of the derived dSNPs, indels and SVs in each group was higher than that in the corresponding sSNPs (Figure 4C), suggesting that deleterious sites were likely to be sheltered in the heterozygous state. 

### 3.5. Deleterious Mutations Were Significantly Correlated with Recombination Rates

**Figure 5:**
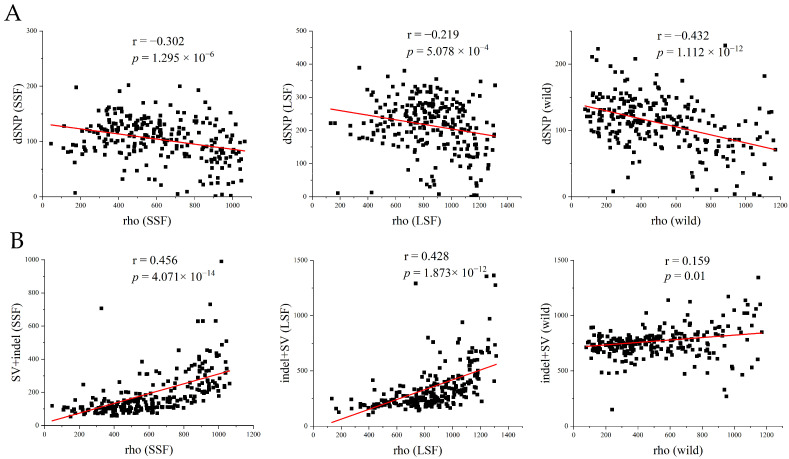
Analysis of genomic variation patterns relative to recombination based on all variant information. (**A**) Number of dSNPs in each window for the three groups (wild, SSF, and LSF). (**B**) Number of SVs in each window for the three groups. The red line represents the regression line, and Pearson’s correlation coefficient and significance *p*-value are annotated in each graph. The recombination rate (rho) and number of variants (isolating + fixed) were measured in 50 kb windows.

To further investigate the relationship between deleterious mutations and the recombination rate, we used FastEPRR to calculate the recombination rate (rho) in 50 kb windows in each group. The results showed that the recombination rate in the LSF group (17.0 rho/kb) and in the SSF group (12.3 rho/kb) was higher than that in the wild population (5.6 rho/kb). The higher recombination rate might have resulted from a higher frequency of outcrossing in the domesticated population. Within the lineage, the recombination rates were lower than 1 rho/kb, and only in Baijiu, beer, wine, Russia, and Malaysia were they above 2 rho/kb (Figure S4). We further analyzed the association between the number of dSNPs and the recombination rate in the wild, LSF, and SSF groups and found that the number of dSNPs was significantly negatively correlated with the recombination rate (Figure 5A), whereas the number of indels/SVs showed a significant positive correlation with the recombination rate (Figure 5B). The main reason for this might be that recombination is an important source of indels and SVs.

### 3.6. Positive Selection Reduced the Enrichment of Deleterious Variants

**Figure 6:**
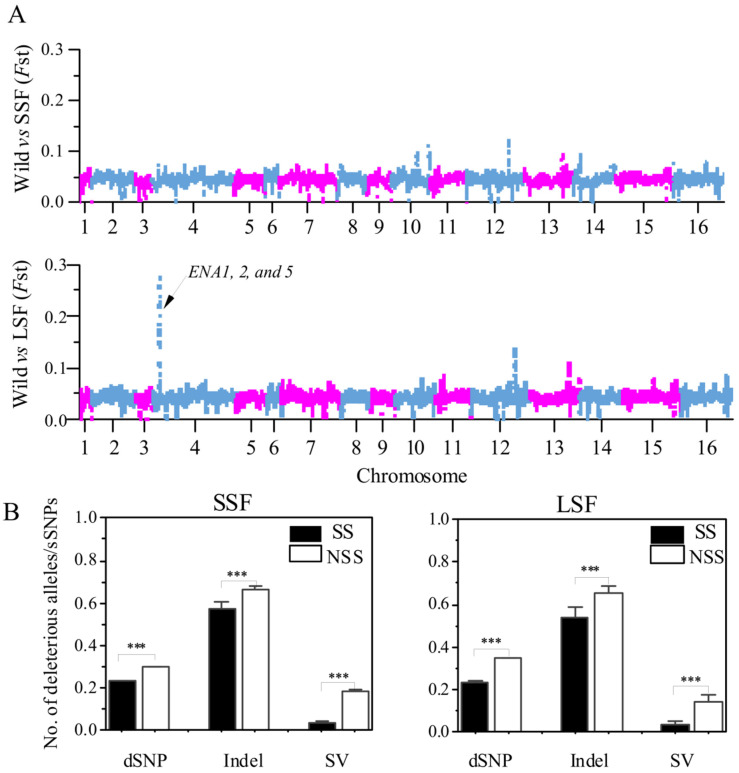
Comparison between selection scan regions (SS) and non-SS regions (NSS) based on the entire variant dataset. (**A**) Manhattan plot of *F*_ST_ values, based on 10 kb window variants is presented for comparisons between wild and SSF strains, as well as between wild and LSF strains. (**B**) Ratios of dSNPs, indel, and SVs to sSNPs in SS and non-SS regions of SSF and LSF strains. Error bars represent mean ± SD. *** *p* = 0.001 (*t*-test).

To determine the relationship between deleterious variants and selected regions, we compared the number of the various mutations in the selected scan regions (SS) and non-selected scan regions (NSS). The SS was determined by *F*_ST_ analyses. Briefly, the Fst analyses in the 10 kb slide windows to analyze the differentiation between the domesticated groups relative to the wild group and the top 1% *F*_ST_ windows were defined as putatively selected scan regions. Based on this method, we identified 320,980 bp and 249,990 bp putative selected regions in the SSF and LSF groups, respectively (Figure 6A). Then, we calculated the ratio of deleterious mutations to sSNPs in each window and found that these ratios in the SS region were significantly decreased compared to that in NSS regions (Figure 6B). In particular, the ratio of SVs/dSNPs in the SS region decreased by 79% and 75% compared to the NSS region in the SSF and LSF groups, respectively. Furthermore, in the LSF groups, we found a 28 kb region in chromosome 4 with the highest *F*_ST_ peak (Figure 6A), which only contains SNP sites. A total of 15 genes were identified and 3 of these genes (ENA 1, 2, and 5) were associated with sodium ion transport (Table S4).

## 4. Discussion

Domesticated *S. cerevisiae* has been shaped for a long time by the emergence of novel and highly specific man-made environments, like food and beverage fermentations [47]. During the domestication process, microbes gained the capacity to efficiently consume particular nutrients, cope with a multitude of industry-specific stress factors, and produce desirable compounds, often at the cost of a reduction in fitness in their original, natural environments. However, few studies have focused on analyzing the genetic load during the domestication of *S. cerevisiae.* In this study, we investigate the genomic patterns of deleterious mutations (dSNPs, indels, and SVs) in *S. cerevisiae* and explore their evolution during domestication.

To accurately capture short-range indels and SVs, we used a pan-genome-based approach, which has been shown in several studies to allow more accurate genotyping of SNPs, short-range indels, and SVs from short-range read data [33,48,49,50]. In our study, we first constructed a pan-genome map using 41 representative high-quality *S. cerevisiae* genomes and the reference genome S288C. This pan-genome map is highly complex, with a size of more than twice that of a single genome. This ratio is much higher than that reported in some studies focusing on pan-genomes of species, such as tomato (*Solanum lycopersicum*) [51] and wheat (*Triticum aestivum*) [52]. For example, 16 high-quality wheat genomes representing the global variation in modern bread wheat cultivars were assembled into a graph pan-genome with a genome size of 15.8 Gb, and approximately 2.0 Gb absent for a single genome [52]. Even for certain genera, such as *Bos* spp. [53] and *Vitis* spp. [54], their pan-genome size was about 1.2- and 1.5-fold that of a single genome. Several reasons may explain the larger size of the *S. cerevisiae* pan-genome. (1) Chromosomal rearrangement and fusion events hinder alignment with reference sequences, resulting in misidentification as new sequences. (2) Domesticated *S. cerevisiae* from special environments have hybrid origins involving horizontal gene transfer from closely related species [23]. (3) An increase in the number of copies in a particular region causes duplicate variants to be mistaken for insertion sequences. (4) Transposon sequences are inserted repeatedly. For example, we identified 6038 structural variants originating from the Ty2 LTR sequence on chromosome 14, aligning with previous findings linking repetitive sequences to Tys, LTRs, and tRNAs [28,55]. 

In our findings, we also discovered some interesting aspects. Overall, the domesticated population has a significantly higher heterozygosity of SNPs compared to the wild population, while the heterozygosity of indels and SVs shows a slight increase (see Figure 2C and Figure 4C). The higher number of SNPs in the domesticated *S. cerevisiae* population can be attributed to their predominant mode of asexual reproduction, allowing them to rapidly accumulate a large number of SNP mutations due to the absence of nutrient limitations. On the other hand, indels and SVs, which could have a significant functional impact, accumulate relatively slowly; this is potentially due to artificial selection and environmental pressures. Additionally, we observed a clear correlation between SVs, indels, and recombination rates in the domesticated population, which is likely a result of multiple rounds of hybridization early in the domestication process, leading to the emergence of new phenotypes along with indels and SVs. In contrast, the wild *S. cerevisiae* population remains predominantly isolated, with rapid population growth occurring only in specific seasons and environmental conditions, without the limitations seen in the domesticated strains [30].

Most population structure studies are currently performed using SNPs; however, SV-based population structure studies are likely to improve our understanding of the adaptation and evolution of species [12,56]. The present study was the first to analyze the indels and SVs in the population of *S. cerevisiae*. Using the PAV of variants across 618 *S. cerevisiae*, we obtained a consistent NJ tree with that obtained using SNPs [24,31]. The high level of heterozygosity of SNPs in domesticated isolates of *S. cerevisiae* was consistent with previous studies [24,57,58]. Compared to the SNPs, the heterozygosity of indels and SVs in the wild isolates was obviously increased. In the lineage TW2, the heterozygous indels accounted for about 0.8, while the ratio of heterozygous SVs in some wild isolates from secondary forests, orchards, fruit, and oak also reached 0.8. The analyses of fitness effects found that the nSNPs, indels, and SVs were predominantly deleterious; in particular, the fitness effects of 90% of the SVs were highly deleterious (fitness < −100). Based on the SIFT score, we determined the dSNPs, and the additive burden of the dSNPs in the domesticated group was significantly higher than in wild groups, reflecting the cost of domestication in *S. cerevisiae*. However, the heterozygosity levels of the dSNPs were significantly higher than those of the sSNPs. Moreover, the levels of dSNPs heterozygosity were positively correlated with its harmfulness. This is not unexpected, because most harmful mutations are at least partially recessive and therefore could only expose their damaging effects in homozygous states [32]. Especially during breeding practices, deleterious mutations in homozygous states are easily observed phenotypically, which promotes purging and breeding decisions, whereas such damaging alleles are masked in heterozygous states and thereby their transmission and accumulation would be facilitated. For budding *S. cerevisiae*, there is an alternative explanation, which is that the ancestor(s) of the domesticated lineages was/were formed by outcrossing between genetically different wild isolates [59]. The heterozygosity of the domesticated isolates is probably maintained due to the loss of sexuality, reduced spore viability, and the advantage of heterosis for living in nutrient rich fermentation environments [9,24,60]. Unlike the SNPs, the additive burden of the indels and SVs in the domesticated groups decreased relative to the wild groups. These differences in the additive burden might be due to the recessive burden. The number of deleterious variations in the SS regions were obviously higher than the NSS region. Furthermore, the number of SVs in the SS regions was about 5-fold higher than that in the NSS regions, reflecting the fact that the SVs suffered more selection. Thus, most deleterious recessive variants tend to be masked in a heterozygous state. This is particularly true of clonally propagated crops [13]. However, highly heterozygous deleterious mutations may severely limit their breeding potential. When *S. cerevisiae* underwent selfing or crossing in breeding, most of the deleterious mutations become homozygous, resulting in severe inbreeding depression, which is also observed in some clonally propagated plants, such as potato [15]. Thus, how to deal with deleterious alleles or reduce the genetic load also needs to be considered during *S. cerevisiae* breeding.

## 5. Conclusions

In summary, this study was the first to utilize *S. cerevisiae* as a model to analyze the cost of domestication in fungi and found that the domesticated isolates undergo a higher additive burden of dSNPs, but a lower burden of indels and SVs. The main reason might be that indels and SVs tend to be heterozygous, and the heterozygosity in the domesticated isolates was obviously higher than that in the wild isolates. The levels of heterozygosity were correlated with the harmfulness of genetic variants. The heterozygous state can alleviate their harmful effects but could constrain their breeding potential. Thus, the deleterious alleles and the genetic load must be considered in further breeding.

## 6. Glossary

Deleterious mutation: mutation in which the protein product of a gene is not produced, is produced but not functional, or is produced and interferes with normal function. Such mutations arise from single base changes or more extensive insertions, deletions, or frameshifts.

nSNPs: Nonsynonymous single nucleotide polymorphisms are single base changes leading to a change in the amino acid sequence of the encoded protein.

sSNPs: Synonymous single nucleotide polymorphisms, which change a nucleotide, but not the encoded amino acid, are perceived as neutral to protein function and thus are classified as benign.

Cost of domestication hypothesis: Increase in the number of deleterious genetic variants, fixed or segregating, in the genomes of domesticated species.

Heterosis: Heterosis, also known as hybrid vigor, refers to the phenomenon where the offspring of two genetically distinct individuals exhibit enhanced or superior traits compared to either of the parents.

Inbreeding depression: Reduced biological fitness of a given population or line because of selfing.

Dominance hypothesis: In genetics and evolutionary biology, it posits that the expression of traits influenced by certain alleles (alternative forms of a gene) is primarily determined by the dominance relationships between these alleles. In simpler terms, it suggests that the phenotype (observable characteristic) expressed by an organism depends on whether the alleles it inherits are dominant or recessive.

Adaptive variation: The genetic variations within a population that provide an advantage under specific environmental conditions. These variations allow individuals with particular traits to survive, reproduce, and pass on those advantageous traits to their offspring. Adaptive variations are key components of natural selection, driving the adaptation of populations to their changing environments over evolutionary time.

Selective sweeps: The process by which a beneficial mutation eliminates or reduces variation in linked neutral sites as it increases in frequency in the population.

## Data Availability

The authors confirm that the data supporting the findings of this study are available within the article its Supplementary Materials.

## Supplementary Materials

The following supporting information can be downloaded at: https://www.mdpi.com/article/10.3390/jof10080575/s1. Figure S1: Based on the NJ tree constructed from variations among 687 strains of yeast, the colors of the labels denote the strains’ origins, while the colors of the branches represent different fermentation states. The blue denotes wild strains, green denotes strains in solid-state fermentation (SSF), and red denotes strains in liquid-state fermentation. Figure S2: The number of SVs in three groups. Figure S3: Analysis of variation hotspots and conserved regions during the domestication process. (A,B): Whole-genome genetic diversity analysis of LSF and SSF groups based on 20kb sliding windows. The red lines indicate the distribution of the top 1% highest pi values, while the black lines indicate the distribution of the bottom 1% lowest pi values. (C,D): Pathway and process enrichment analysis of annotated genes in the top 1% highest pi value regions. (E,F): Pathway and process enrichment analysis of annotated genes in the bottom 1% lowest pi value region. Figure S4: Genome-wide recombination rate analyses of yeast lineages from different sources. Each dot represents the recombination rate rate value (rho/kb) within a 50-kb sliding box. Table S1: The Information of 41 third-generation genomes and the reference genome S288C. Table S2: 687 Saccharomyces cerevisiae subgroups and statistics of various mutation information. Table S3: The ratio of pangenome size/reference size in S. cerevisiae and other published species. Table S4: Functional enrichment and annotation of 15 genes from significantly differentiated regions of difference in LSF strains.
