# Supplementary material for: Graph-Based Pan-Genome Reveals the Pattern of Deleterious Mutations during the Domestication of Saccharomyces cerevisiae
Source: J Fungi (Basel). 2024 Aug 14;10(8):575. doi: 10.3390/jof10080575 (PMC11355510; doi:10.3390/jof10080575)
Supplement: Supplementary file 1 [file jof-10-00575-s001.zip › supplementary figure.pdf]

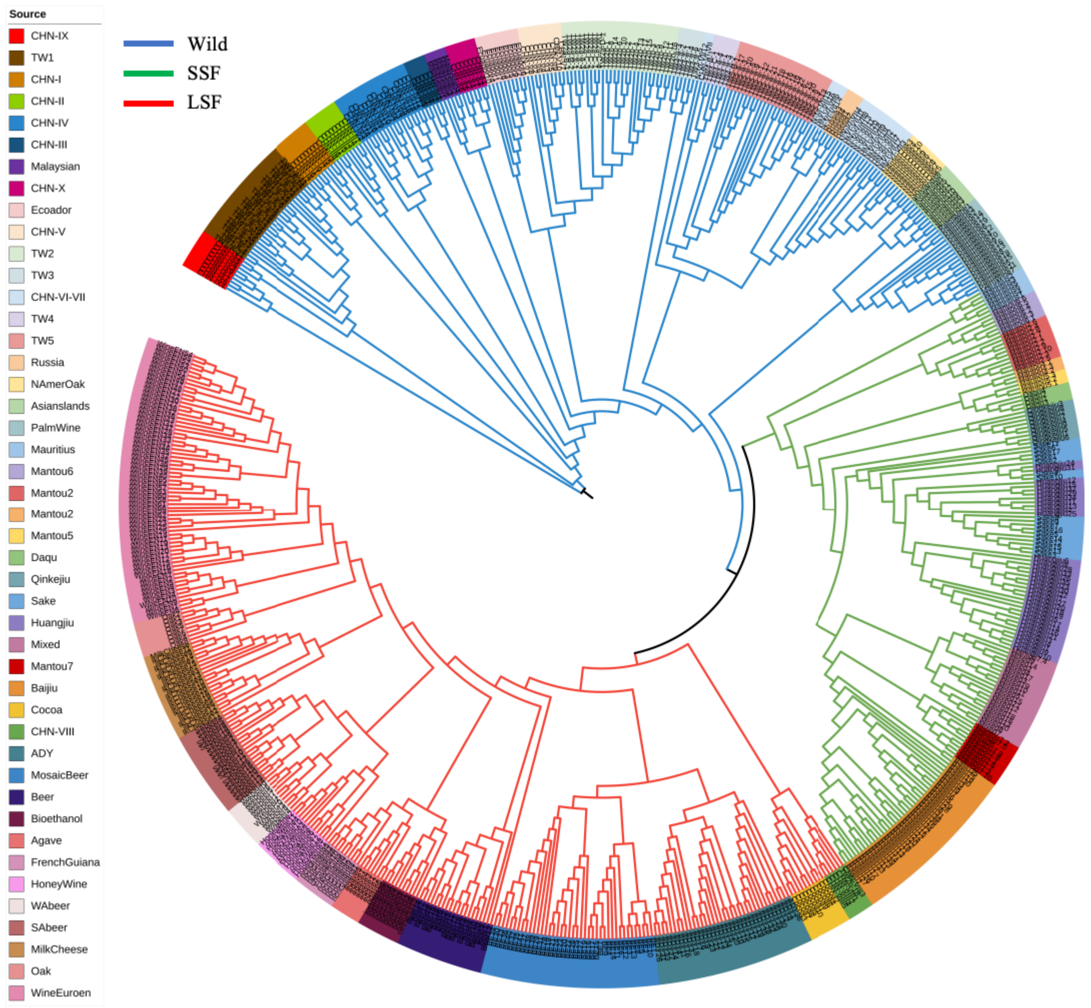

Figure S1: Based on the NJ tree constructed from variations among 687 strains of yeast, the colors of the labels denote the strains' origins, while the colors of the branches represent different fermentation states. The blue denotes wild strains, green denotes strains in solid-state fermentation (SSF), and red denotes strains in liquid-state fermentation.

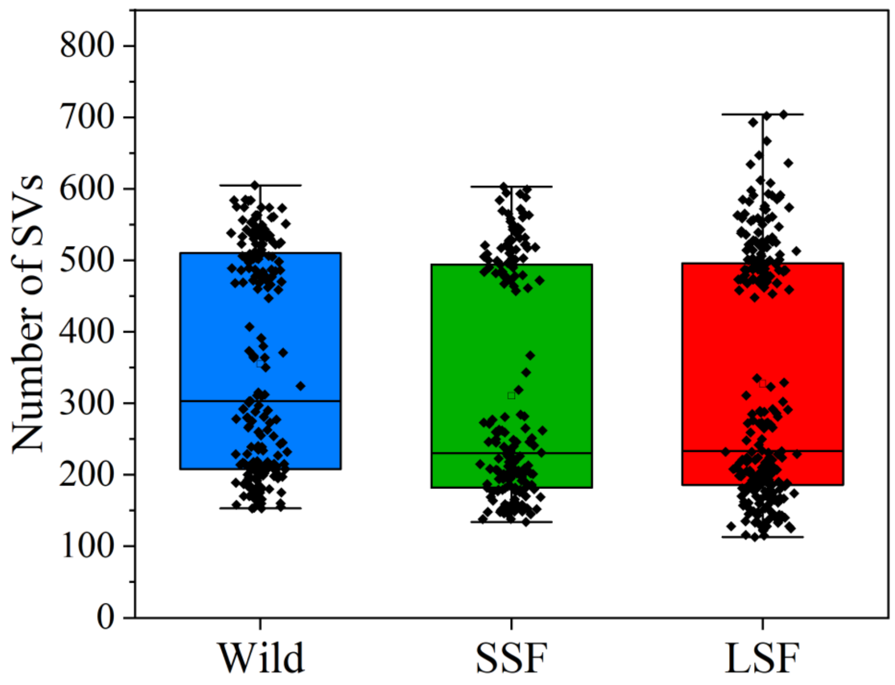

Figure S2: The number of SVs in three groups.

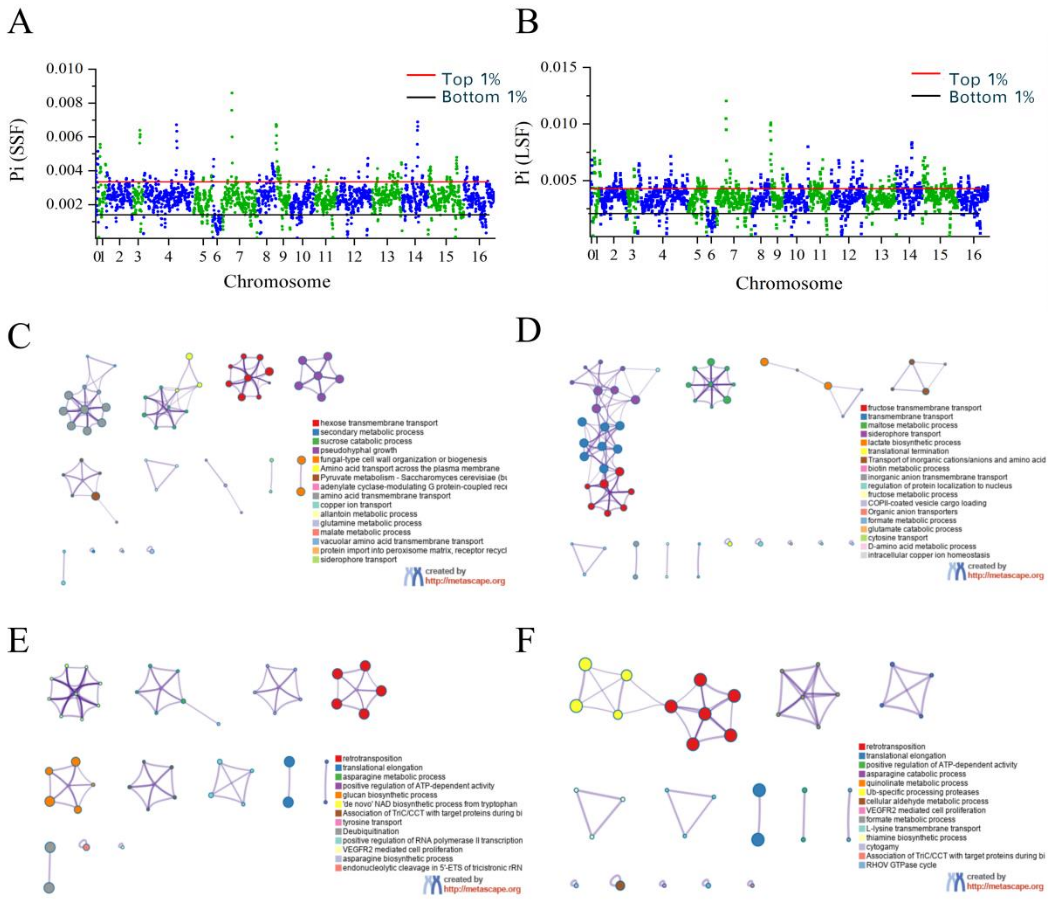

Figure S3: Analysis of variation hotspots and conserved regions during the domestication process.A-B: Whole-genome genetic diversity analysis of LSF and SSF groups based on 20kb sliding windows. The red lines indicate the distribution of the top 1% highest pi values, while the black lines indicate the distribution of the bottom 1% lowest pi values. C-D: Pathway and process enrichment analysis of annotated genes in the top 1% highest pi value regions. E-F: Pathway and process enrichment analysis of annotated genes in the bottom 1% lowest pi value region.

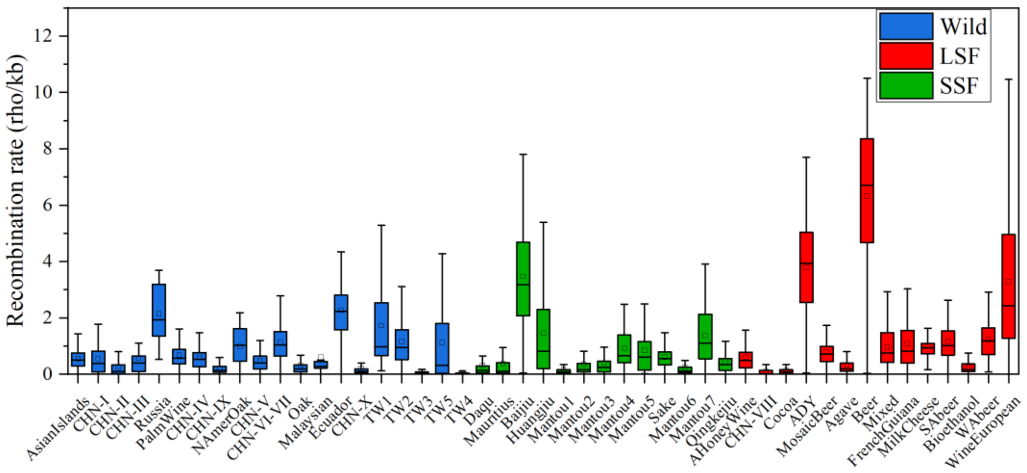

Figure S4: Genome-wide recombination rate analyses of yeast lineages from different sources. Each dot represents the recombination rate rate value (rho/kb) within a 50-kb sliding box.
